# Head-Shaking Nystagmus in Posterior Canal Benign Paroxysmal Positional Vertigo with Canalolithiasis

**DOI:** 10.3390/jcm10050916

**Published:** 2021-02-26

**Authors:** Hyung Lee, Hyun Ah Kim

**Affiliations:** Department of Neurology, School of Medicine, Keimyung University, Daegu 42601, Korea; hlee@dsmc.or.kr

**Keywords:** benign paroxysmal positional vertigo, head-shaking nystagmus, otolith

## Abstract

Background: There have been several studies about head-shaking nystagmus (HSN) in posterior canal benign paroxysmal positional vertigo (PC-BPPV). The purpose of the study was to determine the characteristics of HSN and its relationship with head-bending nystagmus (HBN) and lying-down nystagmus (LDN) in PC-BPPV and to suggest a possible pathomechanism of HSN based on these findings. Methods: During the study period, 992 patients with BPPV were initially enrolled. After excluding horizontal or anterior canal BPPV, multiple canals involvement, secondary causes of BPPV, identifiable central nervous system (CNS) disorders, unidentifiable lesion side, or poor cooperation, 240 patients with unilateral PC-BPPV were enrolled. We assessed the frequency, pattern of HSN, and correlation with other induced nystagmus after positional maneuvers such as head bending, lying down, head-turning, and Dix-Hallpike test. Results: Approximately 32% of patients with PC-BPPV showed HSN. Among patients with HSN, approximately 61% of patients showed predominantly downbeat nystagmus, and two-third of them had a torsional component. The torsional component was mostly directed to the contralesional side. Horizontal nystagmus (36%) and upbeat nystagmus (3%) were also observed after head-shaking in PC-BPPV. The presence of HSN was significantly correlated with that of HBN in PC-BPPV (*p* = 0.00). The presence of a torsional component of HSN was also significantly correlated with that of HBN in PC- BPPV (*p* = 0.00). Discussion: Perverted HSN, a typical sign of central vestibulopathy, is common in posterior canal BPPV and related to HBN. For generating HSN in PC-BPPV, the otolithic movements related to the endolymph dynamics seem to be more important than the velocity storage mechanism.

## 1. Introduction

Testing for head-shaking nystagmus (HSN) has been considered a useful way to detect asymmetry of velocity storage within the vestibulo-ocular reflex that occurs after peripheral or central vestibular lesions. For generating HSN, asymmetric vestibular input during head rotation and asymmetric charging of the velocity storage system are essential [1,2,3]. HSN may be observed in a wide variety of peripheral and central vestibular disorders.

Benign paroxysmal positional vertigo (BPPV) is generated by the movement of free-floating otolithic debris in the endolymph of the semicircular canal (canalolithiasis) or by debris attached to the cupula (cupulolithiasis) [4,5]. There have been several studies [6,7,8,9,10] about HSN in BPPV. We recently reported that HSN was frequently found in patients with horizontal canal BPPV (HC-BPPV) and related to head-bending nystagmus (HBN) and lying-down nystagmus (LDN), which may have been produced by the altered dynamics of the otoconia in BPPV [10]. Therefore, we assumed that HSN in HC-BPPV might be contributed to by the otolith movements related to the altered dynamics of the endolymph. Similar to HC-BPPV, posterior canal BPPV (PC-BPPV) may generate HSN, and it may have a relationship with HBN and LDN because the otolithic debris in the endolymph or attached to the cupula may alter the dynamics of the endolymph or the cupula during horizontal head shaking. Two previous studies [8,9] have described the HSN in PC-BPPV. However, both studies were not focused on PC-BPPV. Furthermore, the detailed data for HSN in PC-BPPV are not shown in the study [8], or their samples were likely to be too small to allow any significant clinical conclusions to be drawn [9]. Additionally, neither study investigated the association of HSN with other induced positional nystagmus such as HBN and LDN. So, we sought to assess the frequency and pattern of HSN in a large number of patients with PC-BPPV. The purpose of the study was to determine the characteristics of HSN and its relationship with HBN and LDN in PC-BPPV and to suggest a possible pathomechanism of HSN based on these findings.

## 2. Experimental Section

### 2.1. Patients

Between January 2012 and February 2014, we identified 992 consecutive patients from the dizziness/vertigo registry at the Keimyung University Dongsan Medical center who had BPPV.

### 2.2. Inclusion and Exclusion Criteria

To determine PC-BPPV, patients were tested using the Dix-Hallpike test. The diagnosis of PC-BPPV was based on the following: (1) a history of brief episodes of positional vertigo, (2) geotropic torsional upbeat nystagmus to one side when tested with the Dix-Hallpike test, which develops with a short latency of several seconds and resolves within 1 min. To exclude patients with underlying peripheral vestibular disorders or central positional nystagmus, all patients also received detailed neurotologic examinations including spontaneous and gaze-evoked nystagmus, horizontal head impulse, horizontal and vertical smooth pursuit and saccades, limb ataxia, and balance function, in addition to routine neurologic examinations. Subjects were excluded if they had any other symptoms and signs of central vestibulopathy or peripheral hypofunction and musculoskeletal or visual impairment. We excluded patients suspicious of vestibular migraine, Meniere’s disease, or other inner ear diseases. Multi-canal BPPV, or cupulolithiatic type of PC-BPPV, which shows more persistent nystagmus (lasting more than 1 min), were also excluded.

### 2.3. Neuro-Otologic Evaluation

Nystagmus was first observed without fixation using a video-Frenzel goggle system (SLMED, Seoul, Korea). Eye movements were also recorded using three-dimensional videooculography (SMI, Teltow, Germany, resolution of 0.1 sampling rate of 60 Hz). Head-turning test, Dix-Hallpike test, head bending test, and lying-down test were performed in order. The affected ear was determined by the direction of Dix-Hallpike test that induces geotropic upbeat nystagmus.

Patients were asked to sit in the head-upright position with their eyes looking forward for 30 s and then to bend the head 60° forward of the pitch axis for 50 s for the observation of nystagmus (HBN) [11]. Thereafter, the patients were asked to take an upright-sitting position with their eyes looking forward for 30 s and then to quickly lie down for 50 s; the nystagmus (LDN) was then examined [12]. HSN was induced using a passive head-shaking maneuver. A positive HSN test result was defined as the presence of at least three beats of nystagmus after 20 cycles of passive head rotation at a rate of about 2 Hz with the head tilted 30 degrees forward in the plane of the horizontal semicircular canal. The presence of HSN was defined only when at least five consecutive nystagmus beats were recorded within 5 s from the end of head-shaking and when the nystagmus lasted more than 5 s. The maximal slow phase velocity (SPV) of the induced nystagmus must exceed 3 degrees per second for horizontal HSN, 2 degrees per second for vertical HSN, and 2 degrees per second for torsional HSN. The detailed testing technique has been published previously [10,13]. The perverted HSN was defined when the induced nystagmus was predominantly vertical or torsional after horizontal head shaking and the velocity of either the vertical or the torsional component was more than 2 degrees per second.

### 2.4. Statistical Analysis

We used a Chi-squared test for comparison of the presence of HSN, HBN, and LDN. We also used the Spearman correlation for evaluating the correlation between the velocity of HSN and HBN or LDN. All analyses was performed using SPSS version 22 (SPSS, Chicago, IL, USA). A statistically significant association between an exposure and the outcome was declared at a *p*-value < 0.05. All of the experiments complied with the tenets of the Declaration of Helsinki, and the study protocol was also reviewed and approved by the Institutional Review Board of Keimyung University Dongsan Medical Center (IRB no. 2020-05-001).

## 3. Results

After excluding horizontal or anterior canal BPPV, cupulolithiatic type of PC-BPPV, multiple canals involvement, secondary causes of BPPV, identifiable central nervous system (CNS) disorders, unidentifiable lesion side, and poor cooperation, 240 patients with PC-BPPV were finally enrolled for this study. Female was predominant (68.5%). The mean age was 59.0 ± 13.0 years. The symptoms duration from attack to the test was 16.2 ± 24.2 days. The lesion side was left ear involvement. (52.5%). Approximately one third (77/240) of patients with PC-BPPV showed HSN. Among patients with HSN, approximately 61% (47/77) of patients showed predominantly downbeat nystagmus. In them, torsional (32/77, 42%) or horizontal (3/77, 4%) components were also recorded. The torsional components were contralesional in 28 (88%) and ipsilesional in four patients. Twenty-eight (28/77, 36%) patients showed pure horizontal nystagmus, in whom 12 had ipsilesional and 16 contralesional horizontal nystagmus. Other (2/77, 3%) showed upbeat nystagmus after head-shaking. Illustrative cases with each pattern of HSN in PC-BPPV are shown in Supplementary Video S1, Video S2 and Video S3. The maximal horizontal SPV of HSN ranged from 5 to 25 degrees/second (mean ± SD = 11.1 ± 5.3 degree/sec). The maximal down and torsional SPV of HSN ranged from 4 to 20 degrees/second (mean ± SD = 8.4 ± 3.7 degree/sec) and from 3 to 14 degrees/second (mean ± SD = 5.3 ± 2.7 degree/sec), respectively. HBN and LDN were observed in 61 (61/240, 25%) and 27 (27/240, 11%) patients with PC-BPPV, respectively. HBN was all predominantly downbeat, and 55 patients (55/61, 90%) had a torsional component. The torsional components were all contralesional. LDN was all upbeat with a torsional component. The torsional components were all ipsilesional. The maximal down and torsional SPV of HBN were 3.7 ± 1.2 degree/sec (3 to 8 degrees/sec) and 3.3 ± 1.2 degree/sec (3 to 10 degrees/sec), respectively. The maximal up and torsional SPV of LDN were 8.5 ± 9.6 degree/sec (3 to 50 degrees/sec) and 8.3 ± 11.4 degree/sec (3 to 60 degrees/sec), respectively. The presence of downbeat HSN was significantly correlated with downbeat HBN (*p* = 0.00), and the presence of torsional HSN was also correlated with torsional HBN (*p* = 0.00) (Table 1). The direction of the torsional component of HSN and HBN was all contralesional except in three patients in PC-BPPV.

## 4. Discussion

In the present study, approximately 32% of patients showed nystagmus after head-shaking and 20% of patients had perverted HSN in PC-BPPV. The presence of downbeat HSN was significantly correlated with that of HBN, and the presence of the torsional component of HSN was also associated with that of HBN. HSN in BPPV might be contributed to by the movement of the otolithic debris related to the alteration of the dynamics of the endolymph.

Two previous studies [8,9] described the HSN in PC-BPPV. One study [8] found that perverted HSN was detected in 11.8% of the patients involving mainly the vertical BPPV, which is below the frequency of perverted HSN in our study. The authors suggested that horizontal head-shaking may excite posterior canals because the vertical semicircular canal is not perfectly vertical, and the perverted HSN can occur by the imbalance of the velocity storage mechanism due to the asymmetric vestibular input from the damaged otolith and/or semicircular canal in BPPV. However, this study included patients with a variety causes of central and peripheral vestibular disorders (not focused on PC-BPPV) and the data for the characteristic (e.q., the number of patients with PC-BPPV and the pattern of perverted HSN) of HSN in BPPV were not shown in the paper. In another study [9], 28% of 46 patients with PC-BPPV showed horizontal HSN after horizontal head-shaking and none of these patients showed perverted HSN after horizontal head-shaking. Both studies did not address the association of HSN with other induced positional nystagmus such as HBN and LDN. To the best of our knowledge, this is the first study to investigate the characteristics of HSN after horizontal head-shaking in a large number of patients with unilateral PC-BPPV with canalolithiasis. Indeed, perverted HSN is not specific for central vestibular disorders and is more common in PC-BBPV than previously though.

**Figure 1:**
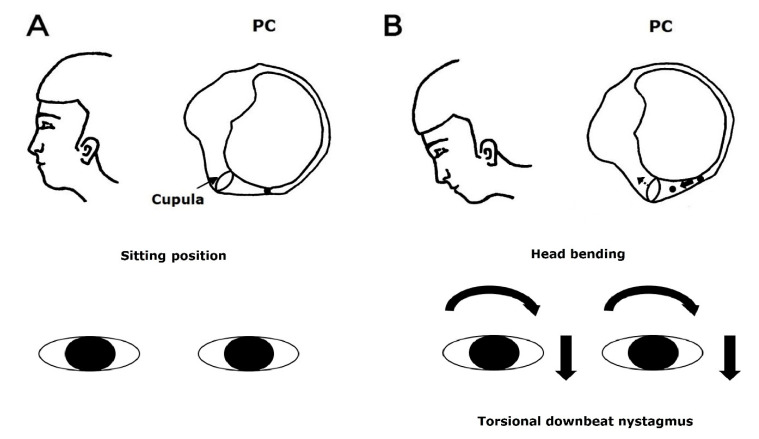
Use of the head bending maneuver to induce nystagmus in benign paroxysmal positional vertigo involving the left posterior canal. The free-floating otolithic debris in the posterior canal at sitting position (**A**) moves toward the cupula, which leads to ampullopetal defection of the cupula during head bending maneuver (**B**). The resulting nystagmus would be downward and torsional, with the upper poles of each eye beating toward the healthy (right) ear.

During the Dix-Hallpike maneuver, the free-floating otolithic debris in the posterior canal moves away from the cupula. It stimulates the posterior canal by inducing ampullofugal flow of the endolymph [14]. Therefore, the resulting nystagmus is upward-beating and torsional with the upper poles of each eye beating toward the affected ear (i.e., the ear in the lower position) [14]. Head bending in PC-BPPV may cause the movement of the otolithic debris in the posterior canal toward the cupula, which leads to ampullopetal defection of the cupula, inhibiting the posterior canal. Accordingly, the resulting nystagmus is downward-beating and torsional with the upper poles of each eye beating toward the healthy ear (i.e., the ear in the upper position) (Figure 1).

Therefore, the direction of nystagmus can be predictable in PC-BPPV. The head-shaking could make a similar movement of the otolithic debris as that during the head bending maneuver, but the detailed mechanism is still uncertain. Our recent study [10] found that HSN is also correlated with HBN in HC-BPPV, and HSN in HC-BPPV might be contributed to by the otolith movements related to the altered dynamic of the endolymph. Similarly, we can presume that the head-shaking itself can produce the movement of otolithic debris in the posterior canal, and it may cause a similar effect in head bending to the posterior semicircular canal based on results of our recent study [10]. The density and viscosity of the otolithic debris, endolymph, and gravity during head oscillation may contribute to the dynamics of the otolith inside the canal and produce HSN. Further studies using an experimental animal model are needed to know the exact mechanism for HSN in PC-BPPV.

Because we could not perform all vestibular work-up and brain images in all patients, we may consider the possibility of central vestibulopathy as a cause of perverted HSN and positional vertigo. However, central vestibulopathies involving the cerebellum and/or brainstem usually present other neurological symptoms and signs such as dysarthria, gait and/or limb ataxia, hemisensory loss, diplopia, or gaze-evoked nystagmus, in addition to positional vertigo/dizziness. Thus, the possibility of central vestibulopathy presenting isolated positional vertigo would be low. Accordingly, a relatively high incidence of perverted HSN in our study cannot be explained by very small portions of focal central vestibulopathies. Indeed, perverted HSN is more common in PC-BPPV than was previously thought. We would like to emphasize the possibility of otolith dynamics within the semicircular canal for perverted HSN in patients with typical PC-BPPV.

## 5. Conclusions

In conclusion, perverted HSN, a typical sign of central vestibulopathy, is common in posterior canal BPPV and related to HBN. For generating HSN in PC-BPPV, the otolithic movements related to the endolymph dynamics seem to be more important than the velocity storage mechanism.

## Figures and Tables

**Table 1:** Comparison of the presence of HBN and LDN in patients with or without HSN in posterior canal benign paroxysmal positional vertigo.

	Presence of All Types of HBN (*n* = 61)	Presence of All Types of LDN (*n* = 27)
Patients with all types of HSN (*n* = 77)	30 (39.0%)	10 (13.0%)
Patients without HSN (*n* = 163)	31 (19.0%)	17 (10.4%)
*p* value	0.00	0.66
	Presence of downbeat HBN (*n* = 61)	Presence of upbeat LDN (*n* = 27)
Patients with downbeat HSN (*n* = 48)	27 (56.0%)	7 (14.6%)
Patients without downbeat HSN (*n* = 192)	33 (17.3%)	20 (10.5%)
*p* value	0.00	0.45
	Presence of torsional HBN (*n* = 55)	Presence of torsional LDN (*n* = 27)
Patients with torsional HSN (*n* = 32)	20 (62.5%)	4 (12.5%)
Patients without torsional HSN (*n* = 208)	35 (16.8%)	23 (11.1%)
*p* value	0.00	0.77

HSN head-shaking nystagmus; HBN = head-bending nystagmus, LDN = lying-down nystagmus.

## Data Availability

The data presented in this study are available on request from the corresponding author. The data are not publicly available due to restrictions of privacy.

## Supplementary Materials

The following are available online at https://zenodo.org/record/4491497#.YDZoHHnRZhE, Video S1. Illustrating case of horizontal head-shaking nystagmus (HSN) in left posterior canal benign paroxysmal positional vertigo (PC-BPPV), Video S2. Illustrating case of pure downbeat head-shaking nystagmus (HSN) in right posterior canal benign paroxysmal positional vertigo (PC-BPPV), Video S3. Illustrating case of torsional downbeat head-shaking nystagmus (HSN) in right posterior canal benign paroxysmal positional vertigo (PC-BPPV).

## References

[1] Hain T., Fetter M., Zee D. (1987). Head-shaking nystagmus in patients with unilateral peripheral vestibular lesions. Am. J. Otolaryngol..

[2] Schmid R., Zambarbieri D. (1991). Oculomotor Control and Cognitive Processes: Normal and Pathological Aspects.

[3] Fetter M.T.S., Koenig E., Dichgans J. (1991). Head-shaking nystagmus: A clinical tool to detect peripheral and central vestibular asymmetries. Oculomotor Control and Cognitive Proceses.

[4] Schuknecht H.F. (1969). Cupulolithiasis. Arch. Otolaryngol. Head Neck Surg..

[5] Hall S.F., Ruby R.R., A McClure J. (1979). The mechanics of benign paroxysmal vertigo. J. Otolaryngol..

[6] Lee S.-U., Kim H.-J., Kim J.-S. (2014). Pseudo-spontaneous and head-shaking nystagmus in horizontal canal benign paroxysmal po-sitional vertigo. Otol. Neurotol..

[7] Kim M.-B., Huh S.H., Ban J.H. (2012). Diversity of Head Shaking Nystagmus in Peripheral Vestibular Disease. Otol. Neurotol..

[8] Yang T., Lee J., Oh S., Kang J., Kim J., Dieterich M. (2020). Clinical implications of head-shaking nystagmus in central and peripheral vestibular disorders: Is perverted head-shaking nystagmus specific for central vestibular pathology?. Eur. J. Neurol..

[9] Lopez-Escamez J.A., Zapata C., Molina M.I., Palma M.J. (2007). Dynamics of canal response to head-shaking test in benign paroxysmal positional vertigo. Acta Oto-Laryngol..

[10] Lee H., Kim H.A. (2020). The association of head shaking nystagmus with head-bending and lying-down nystagmus in horizontal canal benign paroxysmal positional vertigo. J. Vestib. Res..

[11] Lee S.-H., Choi K.-D., Jeong S.-H., Oh Y.-M., Koo J.-W., Kim J.S. (2007). Nystagmus during neck flexion in the pitch plane in benign paroxysmal positional vertigo involving the horizontal canal. J. Neurol. Sci..

[12] Han B.I., Oh H.J., Kim J.S. (2006). Nystagmus while recumbent in horizontal canal benign paroxysmal positional vertigo. Neurology.

[13] Choi K.-D., Oh S.-Y., Park S.-H., Kim J.-H., Koo J.-W. (2007). Head-shaking nystagmus in lateral medullary infarction: Patterns and possible mechanisms. Neurology.

[14] Kim J.-S., Zee D.S. (2014). Benign Paroxysmal Positional Vertigo. N. Engl. J. Med..

